# Population Pharmacokinetics of Rezafungin in Patients with Fungal Infections

**DOI:** 10.1128/AAC.00842-21

**Published:** 2021-10-18

**Authors:** Christopher M. Rubino, Shawn Flanagan

**Affiliations:** a Institute for Clinical Pharmacodynamics (ICPD), Schenectady, New York, USA; b Cidara Therapeutics, San Diego, California, USA

**Keywords:** echinocandin, pharmacokinetics, population pharmacokinetics, pharmacokinetic model

## Abstract

Rezafungin is a novel antifungal agent of the echinocandin class with potent activity against species of *Candida* and Aspergillus, including subsets of resistant strains, and Pneumocystis jirovecii. The objective of this analysis was to develop a population pharmacokinetic (PK) model to characterize the disposition of rezafungin in plasma following intravenous (IV) administration in healthy volunteers and in patients with candidemia and/or invasive candidiasis. The population PK model was based on a previous model from phase 1 data; formal covariate analyses were conducted to identify any relationships between subject characteristics and rezafungin PK variability. A four-compartment model with linear elimination and zero-order drug input provided a robust fit to the pooled data. Several statistically significant relationships between subject descriptors (sex, infection status, serum albumin, and body surface area [BSA]) and rezafungin PK parameters were identified, but none were deemed clinically relevant. Previous dose justification analyses conducted using data from phase 1 subjects alone are expected to remain appropriate. The final model provided a precise and unbiased fit to the observed concentrations and can be used to reliably predict rezafungin PK in infected patients.

## TEXT

Drug pharmacokinetics (PK) and pharmacodynamics (PD) are an ever-important component of modern antimicrobial treatment. Understanding the PK variability of a drug across patient populations is key to drug development and dosing considerations. However, many of today’s antifungal agents and dosing regimens were approved prior to the widespread use of current PK and PD metrics. A growing body of evidence now suggests standard antifungal dosing may be inadequate for certain patient populations and infections (1, 2). PK variability among the triazoles has led to recommendations for therapeutic drug monitoring (TDM) of mold-active agents (3, 4), and dosage adjustments for certain populations (e.g., critically ill patients) may be warranted (2). Echinocandins do not demonstrate PK variability to the extent observed with triazoles and are not recommended for routine TDM (3, 4) yet may be suboptimally dosed in certain populations, such as critically ill patients with invasive candidiasis and patients infected by *Candida* strains with higher MICs (5–7). Critical evaluation of the PK and dosing of current echinocandins in various subpopulations is an area of active research and clinical interest.

Rezafungin is a novel echinocandin antifungal currently in phase 3 development for the treatment of candidemia and/or invasive candidiasis and for single agent prophylaxis against invasive fungal disease caused by *Candida*, Aspergillus, and Pneumocystis species in high-risk patients (8–10). Pharmacokinetic and pharmacokinetic-pharmacodynamic analyses conducted thus far have shown that rezafungin exhibits a long half-life such that front-loaded plasma drug exposures resulting from once-weekly dosing regimens may provide unique PK-PD advantages over other echinocandins (10–13). A population PK model was previously developed for rezafungin using data obtained from healthy volunteers enrolled in two phase 1 studies (12). The model contained four compartments (one central compartment and three peripheral compartments), zero-order drug input via intravenous (IV) infusion, and linear (first-order) elimination. The relative homogeneity of the phase 1 population precluded robust evaluations of the impact of patient characteristics on rezafungin PK. However, the original model included scaling of the model parameters to subject body weight using allometric coefficients (14, 15). This model served as the basis for PK-PD target attainment analyses conducted to identify likely efficacious doses of rezafungin (10, 13).

The analyses reported herein build on the previous population PK analyses by pooling data from phase 1 subjects with data obtained from patients enrolled in a phase 2 study of rezafungin for the treatment of candidemia and/or invasive candidiasis (the STRIVE trial; NCT02734862 [8]). The use of rezafungin PK data from a larger and more diverse population has allowed for the development of a robust population PK model with quantification of the PK of rezafungin in infected patients. In addition, formal covariate analyses have facilitated the identification of patient-specific factors associated with the interindividual variability (IIV) in rezafungin PK.

## RESULTS

### Data.

The final data set consisted of 1,518 rezafungin samples collected from 135 subjects. This included 69 infected patients enrolled in the phase 2 study who provided 416 rezafungin plasma concentrations (six samples per patient) and 66 healthy volunteers enrolled in one of three phase 1 studies with doses ranging from 50 to 1,400 mg IV. Only two concentrations were below the lower limit of quantification. A total of 15 outlier observations were identified during the analysis and excluded from the development of the population PK model (2 observations from phase 1 subjects and 13 observations from phase 2 patients), including one patient who was completely excluded as the overall shape of the rezafungin plasma concentrations versus time did not appear to be consistent with the PK of rezafungin. Most notably, the peak (end of infusion sample) was markedly lower and then increased after the end of the infusion, whereas concentrations were higher and decreased after infusion in all other subjects. The characteristics of subjects included in the final population PK data set are provided in Table 1. The majority of subjects were white/Caucasian (81 to 96% depending on study) and male (46 to 60% depending on study). Subjects enrolled in the phase 1 studies tended to be younger (median age range of 36 to 44.5 years) than those enrolled in the phase 2 study (median age of 57.5 years). Body size measures (e.g., weight, height, and body surface area [BSA]) were relatively consistent across studies. Mean creatinine clearance was lower in subjects enrolled in the phase 2 study as patients with severe renal impairment were eligible for inclusion in that study.

**TABLE 1:** Summary statistics for demographics and clinical laboratory measures among subjects administered rezafungin

Parameter^*a*^	Summary statistics^*b*^			
	Phase 1 studies			Phase 2 study
	SAD study (*n* = 24)	MAD study (*n* = 18)	QTcF study (*n* = 24)	STRIVE (*n* = 69)
Age, yr	42.2 (8.32)	41.1 (9.95)	36 (8.21)	57.3 (14.9)
	41 (25−54)	44.5 (22−54)	36 (20−51)	57.5 (24−88)
Wt, kg	76.4 (9.82)	76 (12.9)	76.2 (13.1)	74.5 (21.3)
	75 (57.8−102)	74.8 (57.1−96.5)	78 (50.3−103)	71 (34–55)
Ht, cm	164 (8.43)	167 (9.16)	169 (10.1)	168 (10.3)
	164 (148−180)	168 (150−187)	166 (155−189)	170 (145−190)
BSA, m^2^	1.83 (0.154)	1.85 (0.197)	1.86 (0.204)	1.83 (0.257)
	1.81 (1.51−2.21)	1.83 (1.58−2.22)	1.83 (1.5−2.3)	1.83 (1.22−2.37)
BMI, kg/m^2^	28.2 (2.71)	27.1 (2.8)	26.7 (3.2)	26.3 (7.74)
	28.5 (22.7−32)	27.2 (22.5−31.7)	26.1 (19.9−31.8)	25.5 (13.9−64.4)
CL_cr_, ml/min/1.73 m^2^	90.2 (12.8)	84.5 (12.5)	94 (17.5)	78.3 (53.4)
	89.8 (62−118)	85.8 (64.5−107)	92.6 (67.2−129)	66.9 (8.57−294)
Albumin, g/dl	4.72 (0.240)	4.74 (0.274)	4.89 (0.275)	2.82 (0.647)
	4.76 (4.34–5.08)	4.76 (4.24–5.08)	4.87 (4.45−5.51)	2.81 (1.62−4.97)
Bilirubin, mg/dl	0.504 (0.21)	0.517 (0.204)	0.492 (0.228)	1.04 (2.19)
	0.5 (0.2−1.1)	0.5 (0.2−0.9)	0.4 (0.2−1)	0.40 (0.07−16.6)
ALT, U/liter	25.3 (10.2)	18.3 (7.05)	21.9 (12.2)	43.7 (45.3)
	24 (11−56)	16.5 (10−34)	17.5 (9−57)	29.0 (6−221)
AST, U/liter	22 (5.86)	18.9 (4.52)	19.8 (7.97)	49.9 (46.1)
	21 (12−32)	18 (13−31)	18 (12−50)	35 (8−247)
				
Race				
White/Caucasian	23/24 (95.8)	15/18 (83.3)	22/24 (91.7)	57/70 (81.4)
Black	0/24 (0)	3/18 (16.7)	2/24 (8.33)	10/70 (14.3)
Other	1/24 (4.17)	0/18 (0)	0/24 (0)	2/70 (2.9)
Not reported	0/24 (0)	0/18 (0)	0/24 (0)	1/70 (1.4)
Sex				
Male	12/24 (50)	10/18 (55.6)	11/24 (45.8)	42/70 (60.0)
Female	12/24 (50)	8/18 (44.4)	13/24 (54.2)	28/70 (40.0)

a BMI, body mass index; CL_cr_, creatinine clearance.

b Summary statistics are shown as mean (standard deviation [SD]) and median (range) for continuous variables and number of individuals/total number of individuals (percentage) for categorical variables.

### Population pharmacokinetic model development.

The structure of the previous population PK model (linear, four-compartment model with zero-order IV input) provided a robust fit to the data and was used as the basis for the covariate analysis. The forward selection process resulted in the inclusion of several statistically significant covariate relationships (all of which were retained in the backward elimination and are described below). Evaluation of the full multivariable model was then performed and resulted in the following modifications to the model:

• Removal of the additive portion of the residual variability model,
• Addition of the covariance between IIV in clearance (CL) and IIV in volume of distribution for the central compartment (Vc),
• Addition of the covariance between IIV in volume of distribution for peripheral compartment 1 (Vp1) and IIV in volume of distribution for peripheral compartment 2 (Vp2),
• Addition of the covariance between IIV in CL and IIV in Vp1.

Backward elimination was then performed using the modified full multivariable model. All of the covariate relationships identified during forward selection remained significant using the stricter criteria for backward elimination. The resultant covariate relationships are described below.

### Final model description and evaluation.

**FIG 1:**
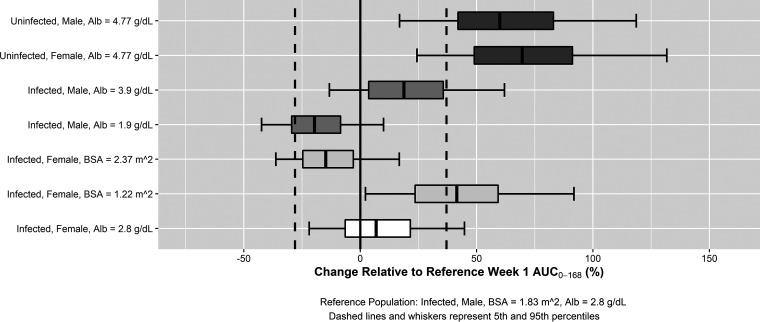
Forest plot showing the impact of statistically significant covariate effects from the final population PK model on rezafungin week 1 plasma AUC_0–168_. Alb, albumin.

The final parameter estimates and their associated precision (percent standard error of the mean [%SEM]) for the final population PK model for rezafungin in healthy volunteers and infected patients are provided in Table 2. The interindividual variability was relatively low for CL and Vp1 (23.7 and 21.7%, respectively) and modest for Vc (38.5%) (16); IIV in Vp2 was estimated relative to the IIV in Vp1. Equations describing the relationship between the population mean PK parameters and statistically significant covariates are provided in Table 2, footnote *a*. The clinical significance of the statistically significant covariates was assessed by evaluating the predicted rezafungin exposure in various subgroups. In Fig. 1, simulated exposures (as assessed by the area under the rezafungin concentration-time curve in the first week [AUC_0–168_]) for the following subject types are shown relative to those of an infected male at median BSA and albumin concentration: healthy male and female subjects at the median serum albumin concentration from the phase 1 studies, infected females at the median serum albumin concentration, and infected patients with BSA and albumin at the 5th and 95th percentiles of patients in the phase 2 study. Predicted rezafungin exposures in females and males were similar in infected or healthy subjects. The largest differences were between healthy and infected subjects; however, these were confounded by albumin differences between the two groups, with all uninfected subjects having albumin concentrations above the 95th percentile of concentrations observed in infected patients (see Discussion). Large increases or decreases in the other two covariates, serum albumin and BSA, did not result in marked differences in exposure as mean exposure values at the 5th and 95th percentile of these values were within twofold of each other and were not considered clinically relevant.

**TABLE 2:** Parameter estimates and standard errors for final population PK model for rezafungin fit to the pooled data from phase 1 and 2 studies^*a*^

Parameter^*b*^	Population mean		Interindividual variability (%CV)		
	Final estimate	%SEM	Final estimate	%SEM	%IIV shrinkage
CL, liter/h	0.254	3.74	0.0562 (23.7)	12.5	3.12
CL-albumin power	−0.844	9.34			
Proportional change in females	−0.133	24.3			
Covariance between CL and Vc	0.0604 (*r*^2^ = 0.4638)	14.1			
Covariance between CL and Vp1	0.0107 (*r*^2^ = 0.043)	51.8			
Vc, liter	11.1	8.87	0.148 (38.5)	10.3	5.74
Vc-BSA slope	7.54	23.6			
Proportional change in infected patients	0.478	27.7			
CLd1, liter/h	18.2	1.09			
Vp1, liter	14.6	4.42	0.0107 (21.7)	22.7	12.4
Vp1-albumin power	−0.829	10.6			
Vp1-BSA power	1.14	15.6			
CLd2, liter/h	0.541	8.11			
Vp2, liter	6.69	11.8			
Vp2-BSA power	2.47	18.4			
Proportional change in infected patients	1.69	19.5			
IIV scaling term relative to Vp1 IIV	1.71	18.8			
CLd3, liter/h	0.0743	7.62			
Vp3, liter	13.6	8.38			
Proportional error, %CV	8.91	2.57			

a Minimum value of the objective function = 621.619. Equations for population mean parameters (SEXF = 1 for females, 0 for males): CL = 0.254(1 − 0.133SEXF)(albumin/4.2)^−0.844^; Vc = [11.1 + 7.54(BSA − 1.83)](1 + 0.478 × infected); Vp1 = 14.6(albumin/4.2)^−0.829^(BSA/1.83)^1.14^; Vp2 = 6.69(1 + 1.69 × infected)(BSA/1.83)^2.47^.

b CL, clearance; CLd1, distributional clearance to peripheral compartment 1.

**FIG 2:**
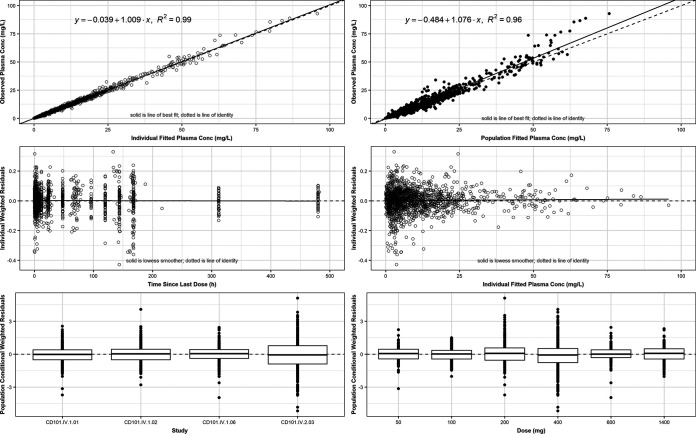
Goodness-of-fit plots for the final population PK model for rezafungin, fit to the pooled data from phase 1 and 2 studies. Conc, concentration.

The model resulted in a precise and unbiased fit to the data as illustrated in the goodness-of-fit plots shown in Fig. 2. The individual- and population-predicted rezafungin concentrations agreed well with the observed concentrations (*r*^2^ = 0.993 and 0.961, respectively; top panels of Fig. 2). In addition, the conditional weight residuals were well balanced relative to the predicted concentration, time since dose, study, and dose administered (middle and bottom panels of Fig. 2).

**FIG 3:**
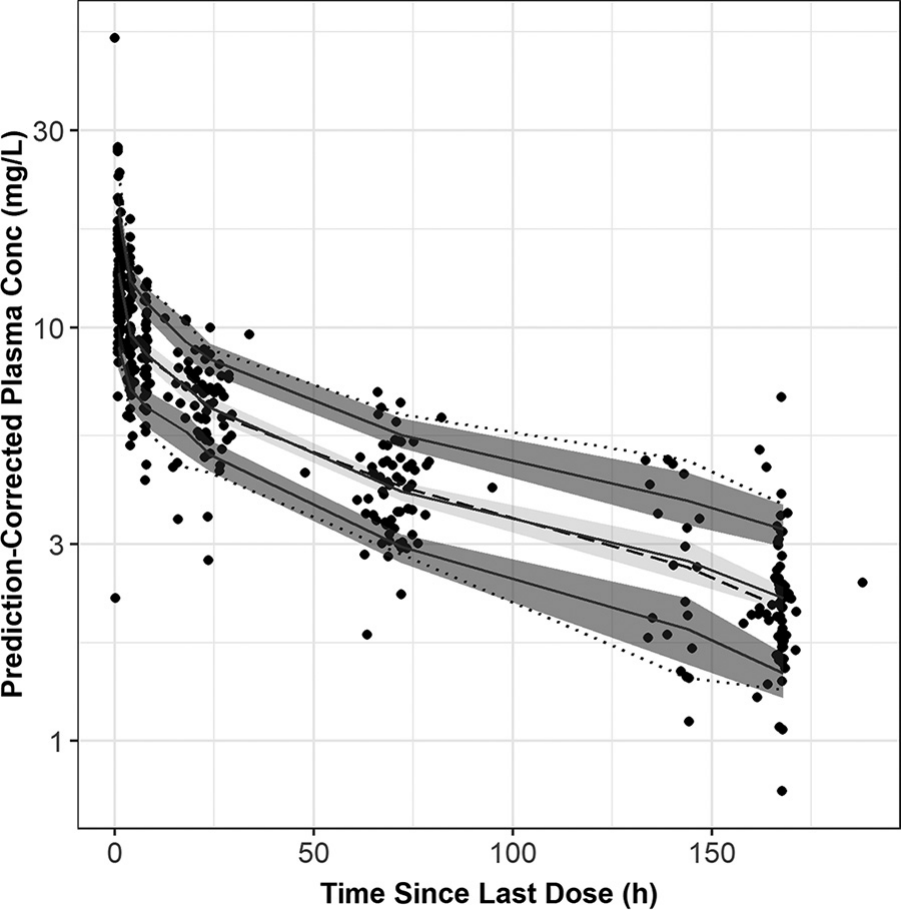
Prediction-corrected visual predictive check plot for data collected from patients enrolled in the STRIVE trial. The dashed line represents the 50th percentile and the dotted lines represent the 5th/95th percentiles of the observed data. The light gray shaded region represents the 95% confidence interval around the 50th percentile of predictions (solid line). The darker gray shaded regions represent 95% confidence intervals around the 5th (lower) and 95th (upper) percentiles of predictions (solid lines show the medians of the respective prediction interval).

The prediction-corrected visual predictive check (PC-VPC) plot for the final model using data from patients enrolled in the STRIVE trial is provided in Fig. 3. The plot indicates that the model is capturing the central tendency of the observed data well, as the median observed concentrations over time universally fall within the 90% prediction interval for the medians from the simulations. The variability is captured less robustly as the model-based simulations tend to underestimate variability in rezafungin concentrations observed in phase 2 patients. The extent of the bias is very small such that the implications, in terms of the assessment of the final population PK model and the expected reliability of future model-based simulations, are negligible.

The estimates of rezafungin PK exposures and key PK parameters obtained from the final model are provided in Table 3.

**TABLE 3:** Summary statistics for rezafungin exposure and PK parameter estimates for patients enrolled in the STRIVE trial

Parameter	Summary statistics^*a*^ for patients in the STRIVE trial (*n* = 68)
AUC_0–168_, mg·h/liter^*b*^	709 (184)
	673 (372–1,210)
*C*_max_, mg/liter^*b*^	16.4 (4.38)
	16.4 (7.10–26.0)
*C*_min_, mg/liter^*b*^	2.07 (0.659)
	1.90 (1.04–3.83)
CL, liter/h	0.365 (0.128)
	0.345 (0.107–0.769)
*V*_ss_, liter	61.0 (26.3)
	52.7 (22.5–158)

a Summary statistics presented as mean (SD) and median (range).

b Rezafungin exposure calculated after first dose and prior to the second dose (i.e., over the 1-week dosing interval).

## DISCUSSION

The population PK model robustly described rezafungin plasma concentration-time data following IV administration of single and multiple rezafungin doses in both healthy volunteers and infected patients. The structure of the previous model (12), a four-compartment model with linear elimination, was retained. The formal covariate analysis resulted in the identification of relationships between multiple subject descriptors (sex, infection status, serum albumin, and BSA) and rezafungin PK parameters that were statistically significant but not likely to be clinically significant. After accounting for the impact of relevant covariate effects, the IIV in rezafungin PK was moderate (23.7% for CL, 38.5% for Vc, and 21.7% for Vp1), and residual variability was estimated to be low (8.91%). The final model provided a precise and unbiased fit to the observed concentrations, and simulation-based diagnostics indicate that the model is appropriately capturing the data such that the future model-based simulations will reliably predict rezafungin PK in infected patients.

Four subject-specific factors were found to be predictive of the variability in rezafungin PK: BSA, infection status, serum albumin, and sex. Consistent with the previous population PK model, which had relationships between body weight and all PK parameters (12), body size in the form of BSA was predictive of the variability in Vc, Vp1, and Vp2. However, body size was not a significant predictor of the variability in rezafungin CL in this population. One possible explanation for the lack of a body size relationship for CL is the fact that sex is predictive of the IIV in CL but none of the volume terms. In this context, sex may be providing a rough surrogate for body size as females tend to be smaller than males. An additional factor is that the variability in CL is largely described by differences in serum albumin such that subjects with low albumin are predicted to have more rapid CL. This is not surprising given only unbound drug is available for removal from the body, and rezafungin protein binding is high (99.2% in rats and 97.4% in humans) (10, 17) with normal serum albumin and decreases with decreasing albumin concentrations. Albumin was also a significant predictor of the IIV in Vp1, suggesting that the amount of unbound drug may also be important in terms of distribution to the peripheral compartment. The inclusion of infection status as a significant predictor of the variability in Vc and Vp1 may reflect some element of confounding insomuch as infected patients had lower serum albumin concentrations relative to healthy volunteers (Table 1). Of note, no relationships were found between renal function and the variability in rezafungin CL, despite the inclusion of patients from the STRIVE trial with creatinine clearance values as low as 9 ml/min/1.73 m^2^ in the analysis data set. This observation suggests that dose adjustment is not necessary in patients with renal impairment.

Despite the relative complexity of the covariate model, none of the identified relationships are likely to be meaningful clinically. As shown in Fig. 1, the impacts of sex, albumin, and body size are limited in the context of the target population of infected patients. The predicted differences between infected patients and healthy subjects, likely due to albumin concentration differences in the two populations, is not anticipated to affect the previous PK-PD target attainment and dose selection results (13). This is due to the counterbalancing effects of lower albumin in infected patients that result in a higher free fraction and a relative lack of change in free-drug AUC, in spite of the lower total AUC estimates. Additional detailed examination of predicted PK-PD target attainment is under way and will be reported separately.

In conclusion, pooling data from healthy subjects and infected patients, including a subset with renal impairment, has resulted in the development of a robust population PK model for rezafungin. Using the model, formal covariate analysis of phase 1 and phase 2 data identified relationships between rezafungin PK and subject factors that were statistically significant but not expected to have clinical significance. A lack of relationship between rezafungin CL and renal function suggests that dose adjustment of rezafungin is not necessary in patients with renal impairment. The final model can be utilized to estimate exposure in patients enrolled in the phase 2 trial and is qualified to conduct Monte Carlo simulations to confirm the selected doses of rezafungin utilized in ongoing phase 3 studies. The final model further contributes to the evidence on rezafungin and the overall development of this novel echinocandin.

## MATERIALS AND METHODS

### Study design.

Data were pooled from three phase 1 studies (NCT02516904, NCT02551549, and CD101.IV.1.06) and one phase 2 study (NCT02734862). The protocols and informed consent were reviewed and approved by an appropriate institutional review board before subject enrollment. The studies were designed and monitored in compliance with the ethical principles of good clinical practice and in accordance with the Declaration of Helsinki.

The first phase 1 study (NCT2516904) was a single ascending dose (SAD) study in which subjects received a single rezafungin dose of 50, 100, 200, or 400 mg or matching placebo administered as a 60-min IV infusion. A total of 32 subjects were enrolled and randomized evenly to each of the four dosing cohorts (6 active and 2 placebo in each cohort). Blood samples for determination of rezafungin plasma concentrations were obtained prior to the dose and at 0.25, 0.5, 1, 1.5, 2, 4, 6, 8, 12, 24, 48, and 120 h after the start of infusion and on days 7, 14 (±1 day), and 21 (±1 day). The second phase 1 study (NCT02551549) was a multiple ascending dose (MAD) study. A total of 24 subjects were enrolled and randomized evenly to one of three cohorts. In cohorts 1 and 2, subjects received 100 or 200 mg of rezafungin (or matching placebo in a 3:1 ratio in each cohort), respectively, as 60-min IV infusions on days 1 and 8. In cohort 3, subjects received 400 mg (or matching placebo in a 3:1 ratio), respectively, as 60-min IV infusions on days 1, 8, and 15. Blood samples were collected predose and at 0.5, 1, 1.5, 2, 4, 6, 8, 12, 24, 48, and 120 h after the start of each infusion and on days 7, 14 (±1 day), 21 (±1 day), and 35 (±1 day, cohort 3 only). The third phase 1 study (CD101.IV.1.06) was designed to assess the impact of single, ascending doses of rezafungin on QTcF interval in healthy subjects. Subjects were randomized to receive either rezafungin (600 or 1,400 mg IV at a rate of 400 mg/h, *n* = 12 per dose group), moxifloxacin (400 mg IV, *n* = 24), or placebo. Only data from subjects randomized to rezafungin were included in the population PK analysis data set. Blood samples for determination of rezafungin plasma concentrations were obtained prior to the dose and at 1.5, 2.5, 3.5, 5, 6, 8, 12, 24, 48, 96, and 168 h after the start of the infusion.

The phase 2 study (NCT02734862, also known as the STRIVE trial [8]) was a multicenter, randomized, double-blind study designed to assess the safety and efficacy of rezafungin, compared to that of caspofungin followed by optional oral fluconazole step-down, in the treatment of patients with candidemia and/or invasive candidiasis. Treatment groups included two dose regimens of rezafungin IV once weekly (400 mg IV or 400 mg IV on week 1 followed by 200 mg on subsequent weeks) or caspofungin IV once daily. Additional doses after day 14 were allowed based on criteria defined in the study protocol. Blood samples for determination of rezafungin plasma concentrations were obtained within 10 min before the end of infusion and either at 4 and 8 h on day 1 (as specified in the original protocol) or from 15 min to 1 h after the end of infusion and 2 to 12 h after the end of infusion on day 1 (as modified by protocol amendment, designed to better inform the population PK model). Additional samples were obtained with random clinical blood draws on day 2 and day 4, as well as prior to the dose on day 8 and day 15 (or day 14 if no day 15 dose was administered).

### Bioanalytical method.

All blood samples were assayed for plasma rezafungin concentration using a liquid chromatography coupled to tandem mass spectrometry (LC/MS/MS) method that has been described previously (11, 12). In brief, the calibration range for the assay was 10.0 ng/ml to 10,000 ng/ml, and interassay accuracy and precision were excellent (accuracy ranged from 0.3 to 4.3%, and precision ranged from 2.7 to 5.0%). The same assay was used for all four studies.

### Population pharmacokinetic modeling.

The previously developed population PK model was a linear, four-compartment model with zero-order IV input and scaling of PK parameters to body weight (12). The structure of this model (i.e., after removal of the scaling of PK parameters to body weight) served as the starting point for model refinement using the pooled data from phase 1 and 2 studies. The actual dates and times of dose administration and PK sample collection were used in the construction of the population PK data set. An outlier was defined as an aberrant observation that substantially deviated from the rest of the observations within an individual. Any suspected outliers were to be tested, and if justified, excluded from this analysis, given the potential for these observations to negatively impact the convergence and/or parameter estimates (18).

Candidate population PK models were fit to the pooled PK data using NONMEM version 7.2, implementing the first-order conditional estimation method with η-ε interaction (FOCE-I) (19). Interindividual variability for each PK parameter was described using an exponential error model assuming a log normal distribution, as feasible. The plasma residual variability was described using a combination additive plus proportional (constant coefficient of variation) error model with alternative structures evaluated as necessary based upon the fit to the pooled data. The following factors were considered when choosing among competing models: the reliability of the structural and statistical parameter estimates, overall reduction in interindividual and residual variability, and the robustness of the fit of the model to the data both visually (using traditional goodness-of-fit plots) and statistically (using the minimum value of the objective function for nested models or Akaike’s information criterion for nonnested models [20]).

A formal covariate analysis was conducted to assess the ability of subject characteristics to explain portions of the interindividual variability for select model parameters. A stepwise forward selection (α = 0.01, 1 df) followed by backward elimination (α = 0.001, 1 df) technique was utilized. Covariates considered included the following: sex, age, weight, body mass index, body surface area, ideal body weight, creatinine clearance (normalized for body surface area), albumin (normalized for variable interlaboratory reference ranges), markers of liver function (alanine transaminase [ALT], aspartate aminotransferase [AST], bilirubin), race, and infection status (defined as “healthy” [phase 1 subjects] or “infected” [phase 2 patients who were enrolled with a systemic fungal infection]).

The final population PK model was qualified by performing a PC-VPC, which is a simulation-based model diagnostic/qualification tool that allows for the comparison of model fit across different dosing regimens and accounts for differences among patient descriptors (21). The procedure used the original analysis data set as a template to simulate PK data for the same number of subjects in 100 new data sets, with each data set featuring the same study design and data collection scheme. The PC-VPC controls for the imbalance in study design across studies by normalizing both the observed and simulated concentration-time data by the median population predictions during each time interval. Summary measures of the distribution (medians and 90% prediction intervals) of predictions and observations were compared visually to assess the model fit.

Patient-specific estimates of rezafungin exposure (AUC from zero to tau [AUC0−τ], maximum concentration of drug [*C*_max_], and minimum concentration of drug [*C*_min_]) were derived using individual *post hoc* PK parameter estimates obtained from the final model. The PK parameters and the mrgsolve package in R (22) were then used to simulate predicted plasma rezafungin concentration-time data for each subject for the duration of time between the first and second doses. *C*_max_ and C_min_ were defined as the highest and lowest predicted concentrations over that first dosing interval, respectively, while AUC_0-_*_τ_* was calculated via numerical integration of the concentration-time profile. Summary statistics for the rezafungin exposures and key PK parameters (i.e., CL and volume of distribution at steady state [*V*_ss_]) were calculated for the patients from the STRIVE trial.

## References

[1] Lepak AJ, Andes DR. 2014. Antifungal pharmacokinetics and pharmacodynamics. Cold Spring Harb Perspect Med 5:a019653. doi:10.1101/cshperspect.a019653. 25384765 PMC4448584

[2] Pea F, Lewis RE. 2018. Overview of antifungal dosing in invasive candidiasis. J Antimicrob Chemother 73:i33–i43. doi:10.1093/jac/dkx447. 29304210

[3] Ashbee HR, Barnes RA, Johnson EM, Richardson MD, Gorton R, Hope WW. 2014. Therapeutic drug monitoring (TDM) of antifungal agents: guidelines from the British Society for Medical Mycology. J Antimicrob Chemother 69:1162–1176. doi:10.1093/jac/dkt508. 24379304 PMC3977608

[4] John J, Loo A, Mazur S, Walsh TJ. 2019. Therapeutic drug monitoring of systemic antifungal agents: a pragmatic approach for adult and pediatric patients. Expert Opin Drug Metab Toxicol 15:881–895. doi:10.1080/17425255.2019.1671971. 31550939

[5] Bader JC, Bhavnani SM, Andes DR, Ambrose PG. 2018. We can do better: a fresh look at echinocandin dosing. J Antimicrob Chemother 73:i44–i50. doi:10.1093/jac/dkx448. 29340605

[6] Bader JC, Lakota EA, Bhavnani SM, Ambrose PG. 2016. Emerging Candida glabrata resistance and echinocandin dosing: a call to arms! Open Forum Infect Dis 3:S515. doi:10.1093/ofid/ofw172.1521.

[7] Sinnollareddy MG, Roberts JA, Lipman J, Akova M, Bassetti M, De Waele JJ, Kaukonen KM, Koulenti D, Martin C, Montravers P, Rello J, Rhodes A, Starr T, Wallis SC, Dimopoulos G, DALI Study authors. 2015. Pharmacokinetic variability and exposures of fluconazole, anidulafungin, and caspofungin in intensive care unit patients: data from multinational Defining Antibiotic Levels in Intensive care unit (DALI) patients Study. Crit Care 19:33. doi:10.1186/s13054-015-0758-3. 25888060 PMC4335513

[8] Thompson GR, III, Soriano A, Skoutelis A, Vazquez JA, Honore PM, Horcajada JP, Spapen H, Bassetti M, Ostrosky-Zeichner L, Das AF, Viani RM, Sandison T, Pappas PG. 2020. Rezafungin versus caspofungin in a phase 2, randomized, double-blind study for the treatment of candidemia and invasive candidiasis: the STRIVE trial. Clin Infect Dis doi:10.1093/cid/ciaa1380. PMC8662762 32955088

[9] Miesel L, Lin KY, Ong V. 2019. Rezafungin treatment in mouse models of invasive candidiasis and aspergillosis: insights on the PK/PD pharmacometrics of rezafungin efficacy. Pharmacol Res Perspect 7:e00546. doi:10.1002/prp2.546. 31763045 PMC6864408

[10] Flanagan S, Sandison T, Locke JB, Ong V, Ye G, Bartizal K, Daruwala P. 2018. CD101 prophylactic dose rationale for prevention of Aspergillus, Candida, and Pneumocystis infections. Biol Blood Marrow Transplant 24:S389–S390. doi:10.1016/j.bbmt.2017.12.484.

[11] Sandison T, Ong V, Lee J, Thye D. 2017. Safety and pharmacokinetics of CD101 IV, a novel echinocandin, in healthy adults. Antimicrob Agents Chemother 61:e01627-16. doi:10.1128/AAC.01627-16. 27919901 PMC5278714

[12] Lakota EA, Ong V, Flanagan S, Rubino CM. 2018. Population pharmacokinetic analyses for rezafungin (CD101) efficacy using phase 1 data. Antimicrob Agents Chemother 62:e02603-17. doi:10.1128/AAC.02603-17. 29555631 PMC5971566

[13] Bader JC, Lakota EA, Flanagan S, Ong V, Sandison T, Rubino CM, Bhavnani SM, Ambrose PG. 2018. Overcoming the resistance hurdle: pharmacokinetic-pharmacodynamic target attainment analyses for rezafungin (CD101) against Candida albicans and Candida glabrata. Antimicrob Agents Chemother 62:e02614-17. doi:10.1128/AAC.02614-17. 29555634 PMC5971579

[14] Mordenti J. 1986. Man versus beast: pharmacokinetic scaling in mammals. J Pharm Sci 75:1028–1040. doi:10.1002/jps.2600751104. 3820096

[15] Holford NHG, Anderson BJ. 2017. Allometric size: the scientific theory and extension to normal fat mass. Eur J Pharm Sci 109S:S59–S64. doi:10.1016/j.ejps.2017.05.056. 28552478

[16] Al-Sallami HS, Cheah SL, Han SY, Liew J, Lim J, Ng MA, Solanki H, Soo RJ, Tan V, Duffull SB. 2014. Between-subject variability: should high be the new normal? Eur J Clin Pharmacol 70:1403–1404. doi:10.1007/s00228-014-1740-8. 25187338

[17] Ong V, James KD, Smith S, Krishnan BR. 2017. Pharmacokinetics of the novel echinocandin CD101 in multiple animal species. Antimicrob Agents Chemother 61:e01626-16. doi:10.1128/AAC.01626-16. 28137817 PMC5365648

[18] Food and Drug Administration, U.S. Department of Health and Human Services. 2019. Guidance for industry: population pharmacokinetics. Food and Drug Administration, Silver Spring, MD.

[19] Bauer RJ. 2017. NONMEM users guides; introduction to NONMEM 7.4.1. ICON Plc, Gaithersburg, MD.

[20] Akaike H. 1979. Bayesian extension of the minimum AIC procedure of autoregressive model fitting. Biometrika 66:237–242. doi:10.1093/biomet/66.2.237.

[21] Bergstrand M, Hooker AC, Wallin JE, Karlsson MO. 2011. Prediction-corrected visual predictive checks for diagnosing nonlinear mixed-effects models. AAPS J 13:143–151. doi:10.1208/s12248-011-9255-z. 21302010 PMC3085712

[22] Baron KT. 2017. mrgsolve: simulate from ODE-based population PK/PD and systems pharmacology models. R package version 0.8.6.

